# Evaluating the results of resistance training using ultrasound or flexed arm circumference: A case for keeping it simple?

**Published:** 2020-08-08

**Authors:** Paulo Gentil, Emily Budzynski-Seymour, Daniel Souza, James Steele, James P. Fisher, Martim Bottaro

**Affiliations:** ^1^College of Physical Education and Dance, Federal University of Goias, Goiania, GO, Brazil; ^2^School of Sport, Health, and Social Sciences, Solent University, Southampton, UK; ^3^Ukactive Research Institute, London, UK; ^4^College of Physical Education, University of Brasília, Brasília, DF, Brazil

**Keywords:** hypertrophy, anthropometry, resistance training, muscles

## Abstract

**Background:**

The present study aimed to compare changes in muscle size when measured by ultrasound (US) muscle thickness (MT) and arm circumference (AC) using data from young men.

**Methods:**

The investigation involved data from three previous studies involving a total of 67 young men who performed resistance training (RT) for 10-12 weeks. Before and after the training period, elbow flexor MT was evaluated by US and AC was measured. We conducted two-stage individual patient data random-effects meta-analyses using both Frequentist and Bayesian hypothesis testing. One-sample analyses examined the absence or presence of a change in both MT and AC, and paired analyses examined whether these differed from one another or equivalent.

**Results:**

One-sample analysis supported that both AC (+4.9%; tp=0.0002; BF10=6,255,759,515) and MT (+3.9%; *P*<0.0001; BF10=7,958,241,773) suggested that change in muscle size had occurred. Frequentist paired comparisons suggested that the estimates of change between both AC and MT measures did not significantly differ (*P*=0.1092), but were not statistically equivalent. Bayesian paired comparisons, however, suggested that MT estimates where greater in magnitude than AC estimates for change in muscle size (BF10=16.39174).

**Conclusion:**

Both MT and AC are able to detect RT-induced changes in muscle size of the upper arm, but that the magnitude of changes may differ. Thus, care should be taken when comparing or combining estimates using either approach.

**Relevance for patients:**

The use of AC might be considered as a practical and low-cost alternative to detect changes in muscle size.

## 1. Introduction

Skeletal muscle mass is considered of critical importance for the preservation of metabolic health and independent locomotion [1-3]. Moreover, there is also great interest in increasing skeletal muscle mass with the aim of maximizing physical performance and for esthetic purposes with many citing it as the primary reason for engagement in resistance training (RT). Considering the importance of muscle mass, RT is used by many individuals as a means to promote skeletal muscle hypertrophy [4]. In this regard, different methods are used to measure muscle size both at single- and across multiple- time points to determine change. These include bioelectrical impedance analysis, dual-energy X-ray, computed tomography, magnetic resonance imaging (MRI), and B mode ultrasound (US).

Recently, the US has become a more popular approach to measurement primarily due to its ease of the use and low-cost [3]. Indeed, it has been shown to track changes in muscular hypertrophy similarly well when compared to MRI providing similar conclusions in the presence of change [5,6], despite not necessarily showing agreement with respect to the magnitude of that change estimate [6]. However, even measures of muscle thickness (MT) using the US may be prohibitive with respect to costs, at least in comparison to another widely used approach: Arm circumference (AC).

MT has gained popularity, especially in the scientific literature [7]; however, AC is still a popular and reliable method for estimating changes in muscle size during RT [8-14]. Indeed, simple anthropometric measurements have been shown to be able to detect the presence of a change in muscle size similarly well compared to MRI [15]. To the best of our knowledge, the US and anthropometric measures have only been examined at single time points where they have been shown to have good relationships and comparative reliability [16]. However, considering the wide use and acceptance of both MT and AC measures by the scientific community, it is not known if the results obtained from them similarly enable conclusions to be drawn about the absence or presence of changes in muscular size, nor whether these estimates are equivalent in magnitude. Considering that the agreement between measures to estimate changes in muscle size is an important question to be asked, particularly with the popularity of combining different methods of measurement in combined estimates within meta-analyses [17], we present this technical note that compared changes in MT and AC using retrospectively collected data from young men undergoing RT.

## 2. Materials and Methods

### 2.1. Participants

Data from 67 young men (both trained and untrained) that participated in the previous studies [18-20] were used in the current analysis. The volunteers were instructed not to change their nutritional habits during the study period, all of them verbally confirmed that they maintained their diet throughout the trial period and no relevant change was reported (i.e., becoming vegetarian, restricting calories, taking nutritional supplements, or ergogenic aids). The experiments were performed in accordance with the ethical standards of the Helsinki Declaration, the protocol was approved by the relevant ethics committee, and the participants signed an informed consent form. Descriptive characteristics of each study are presented in Table 1.

**Table 1:** Descriptive data (Mean±SD).

	Gentil *et al*., 2013	Gentil *et al*., 2015	Gentil *et al*., 2018
Age (years)	22.8±2.7	23.9±3.8	22.3±2.0
Body height (cm)	175.7±7.6	174.1±6.8	177.5±5.1
Body mass (kg)	71.3±9.0	73.4±10.2	80.0±12.4
Muscle thickness (mm)				
Pre	32.9±4.7	32.9±4.6	36.4±4.5
Post	34.9±4.2	34.3±4.2	37.1±4.6
Flexed arm circumference (cm)			
Pre	31.3±2.8	33.0±2.7	36.1±2.6
Post	32.8±2.4	34.3±2.5	36.5±2.8

### 2.2. MT

MT of the elbow flexors was measured before and after the training period using B-mode US (Philips-VMI, Ultra Vision Flip, and model BF). The tests were conducted 3-5 days after the last training session to limit the influence of acute swelling on measurement. During this time, participants were oriented not to participate in any other type of exercise or intense activity. All measurements were conducted using the right arm, at the same time of the day, and the participants were oriented to hydrate normally 24 h before the tests. MT was measured as the distance from the subcutaneous adipose tissue-muscle interface to the muscle-bone interface at 10 cm from the cubital fossa, as previously described [18-20]. The same trained technician performed pre- and post-measurements. The baseline test and retest intraclass correlation coefficient (ICC) for elbow flexors MT varied between 0.93 and 0.98 for the included studies.

### 2.3. Flexed AC

AC was measured on the right-side arm immediately after the MT measures. The arm was raised to a horizontal position in the sagittal (forward) plane, with the elbow at 90°. The subject maximally contracted the elbow flexors, and the largest circumference was measured. Three measures were taken and the average of the values was used during the analyses. The baseline test and retest ICC for AC were 0.96. We acknowledge that the use of a measure at the same point of MT in the relaxed state would provide greater similarity between the tests. However, the study was not aiming to find equivalent measures, but to compare, the results obtained from a measure commonly used in researches to one used in real-world settings. For this reason, we opted to use flexed AC.

### 2.4. Resistance training

Since the data were obtained retrospectively from the previous studies, training was not standardized. In general, all participants trained under the direct supervision of at least one supervisor per five trainees [21] and attended at least 80% of the training sessions [22]. The protocols involved 6-12 weekly sets for the elbow flexors and extensors performed 1 or 2 times per week with 8-12 maximum repetitions per set. To maintain performance in the target repetition range, the loads were reduced if the participant was unable to perform at least eight repetitions and they were increased if it was possible to perform more than 12 repetitions. The full details of the training protocols for each study can be seen in the methods of the original publications [18-20].

### 2.5. Statistical analysis

Due to the different characteristics of the included studies, such as participant sample and specific characteristics of the training interventions, we used a two-stage meta-analytic approach. For Stage 1, the pre-post delta (i.e., change) was calculated and expressed as a percentage for both AC and MT for all participants and means and standard deviations calculated for each study. Stage 2 involved performing random-effects meta-analyses across the studies to test the hypotheses detailed below. A combination of both Frequentist and Bayesian hypothesis testing was conducted and compared here in part to examine the robustness of conclusions drawn to the analysis approach used. All analyses were conducted in R (version 3.6.2; R Core Development Team) using the “metafor” and “BayesFactor” packages using the RMA and meta-test BF functions, respectively. Frequentist and Bayesian one sample meta-analyses were performed to examine whether both AC and MT would yield similar conclusions regarding the absence or presence of change (i.e., whether the change was =0 or >0). In addition, both Frequentist and Bayesian paired comparison meta-analyses were performed to test whether the estimates of change for both AC and MT differed from one another (i.e., whether the difference in change was =0 or >0). In the case that the Frequentist paired comparison was non-significant, equivalence was examined using the two one-sided test (TOST) approach and inspection of whether 90% confidence intervals fell within equivalence bounds set based on measurement error at half the minimal detectable change (MDC) of the measure with the greatest variability. Further, Bayesian interval estimates were also compared to this. Estimates using standardized effect sizes were calculated from both Frequentist and Bayesian meta-analyses for qualitative comparison and to reflect the calculation of standardized effect sizes (Cohen’s *d* – calculated using the % change standard deviation as the denominator for one-samples analyses and pooled % change score standard deviation as the denominator for paired analyses) from different studies using different methods. In the case that the Frequentist paired comparison was non-significant, equivalence was examined using the TOST approach and inspection of whether 90% confidence intervals fell within equivalence bounds set based on measurement error at half the MDC of the measure with the greatest variability. Further, Bayesian interval estimates were also compared to this. The MDC was converted to an upper and lower bound for Cohen’s *d* of -0.02-0.02. Finally, data were presented graphically for visual interpretation using a scatter plot with individual data from each study coded. Frequentist analysis was performed with an alpha threshold set at 0.05 for rejection of the null hypothesis (i.e., μ=0). Bayesian analysis was performed using a default Cauchy before 0.707 centered on zero and Bayes factors (BF10) were calculated and interpreted at either providing evidence for BF10 <0.33 or against (BF10 >3.0) the null hypothesis.

## 3. Results

The Frequentist one sample meta-analyses suggested that significant change had occurred for both AC (*P*=0.0002; I2=67.41%; Q(2)=6.295, *P*=0.043); and MT (*P*<0.0001; I2=0.0%; Q(2)=1.5616, *P*=0.458). Similarly, the Bayesian one sample meta-analyses also provided evidence against the null hypothesis supporting that change had occurred for both AC (BF10=6,255,759,515) and MT (BF10=7,958,241,773). In comparing the estimates of change between both AC and MT measures, the Frequentist paired meta-analysis suggested that these did not significantly differ (*P*=0.1092; I2=0.0%; Q(2)=0.2291, *P*=0.8918). Equivalence was, however, not confirmed as the confidence intervals for the effect estimate exceeded the lower equivalence bound (90% confidence intervals for d=−0.56-0.01). The Bayesian meta-analysis provided evidence against the null hypothesis, thus, suggesting the two estimates differed (BF10=16.39174) and indeed the credible intervals fell outside the equivalence bounds (95% credible intervals for d=0.22-0.89) suggesting that greater estimates occurred with MT.

Cohen’s *d* point estimates, 95% confidence intervals from Frequentist analyses, and Cohen’s *d* empirical mean point estimated and 95% credible intervals from the posterior distribution plot (1000 iterations) from Bayesian analysis for the changes in MT and AC presented in Table 2. When expressed as standardized effect sizes, there were qualitatively similar magnitudes of change observed (Table 2), though, for paired comparisons, these differed between Frequentist and Bayesian analyses.

**Table 2:** Point and interval estimate for Cohen's *d* from Frequentist and Bayesian random-effects meta-analysis models.

Outcome measure	Frequentist model *d* [95% confidence intervals]	Bayesian model *d* [95% credible intervals]
MT change	1.03 [0.73-1.33]	1.08 [0.80-1.37]
AC change	1.03 [0.49-1.58]	1.08 [0.73-1.35]
MT minus AC difference	0.23 [-0.62-0.06]	0.54 [0.22-0.89]

MT: Muscle thickness, AC: Arm circumference

**Figure 1:**
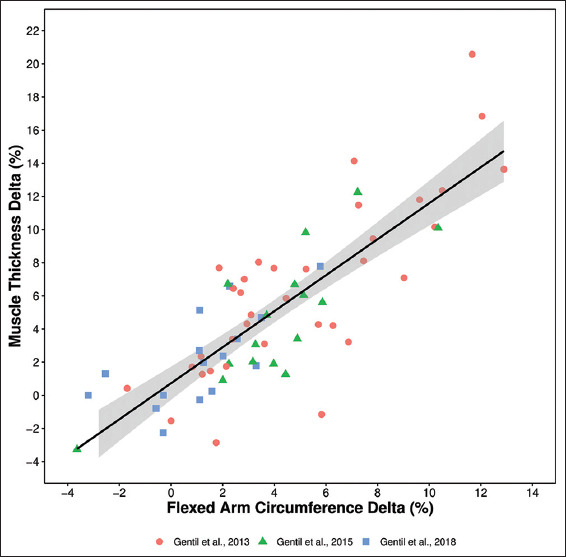
Scatter Plot with study coding. Gentil *et al.*, 2013, (●); Gentil *et al.*, 2015, (■); Gentil *et al.*, 2018, (▲).

Figure 1 presents a scatter plot for the pre-post deltas for AC and MT with each study color coded, along with Pearson’s correlation coefficient for the combined sample.

## 4. Discussion

The purpose of the present study was to investigate the relationship between estimates of changes in muscle size as measured by two popular methods, MT and AC. Frequentist and Bayesian analyses were performed to examine whether both AC and MT would yield similar conclusions regarding the absence or presence of change. Our results suggested that, irrespective of the statistical approach taken, both MT and AC led to the conclusion that changes in muscle size had occurred. However, though Frequentist analyses were unclear as to whether estimates were statistically significantly different or equivalent to one another, Bayesian analyses suggested that the magnitude of estimates of change may be higher for MT. Despite the relationship between changes measured using either MT or AC (i.e., those with larger changes in one typically also experience large changes in the other), the degree of agreement between the two measurement approaches in terms of the magnitude of estimates they provide is less clear. This may have implications, particularly for comparison of magnitudes of changes between studies and in the calculation of summary estimates in meta-analyses using different measurement approaches, something which has been previously commented on [17]. Although it should be noted that, at least for within-measurement effects, standardized effect sizes may show similar magnitudes of change for both methods, and thus, combination in this manner may be best for meta-analyses.

It is important to note that, while both methods seem able to detect changes in muscle size, each has some important differences. For example, in the studies included, MT was specific to the elbow flexors, while AC also involves elbow extensors. The previous studies have shown that different muscles might experience different patterns of hypertrophy with time in response to RT [23]; however, this has not been evaluated between elbow extensors and flexors, the previous studies showed that the mean change in MT was similar between them [24,25]. Another possible source of the disagreement between percentage changes from each measure is that AC does not consider the possible influence of subcutaneous fat, while MT involves only muscle. This might be a limitation when studying people with high levels of fat and also in long-term studies or studies that involve weight loss. In this case, changes in subcutaneous fat might be more evident, leading to an underestimation of changes in muscle hypertrophy while using AC. Measurements at single time points and where AC has been supplemented with measurement of skinfolds to yield estimated arm muscle area independent of subcutaneous fat have been shown to have good relationships and comparative reliability to measurements of MT [16]; however, the relationships were strongest when the total MT of both elbow flexors and extensors was summed. AC is likely a better indicator of total upper arm muscle size, and future studies should examine changes in this outcome with measurement of skinfolds in comparison to total upper arm MT.

From a practical standpoint, the choice of testing modalities should take into account the logistical possibilities. AC has the advantage of requiring minimal equipment, involve simpler procedures, and require less training than MT. On the other hand, MT has the advantage to allow the analysis of single muscles and exclusion of subcutaneous fat from the analysis. Of course, we should note that test-retest reliability is of great importance, so the skill required to take the measurements should not be assumed. The practice of both measurement types is skill dependent and so to enhance the accuracy of measurement practitioners and researchers should be well-rehearsed. One important limitation of the present study is that the analysis was limited to the upper arm and was performed in young eutrophic men. Therefore, the results might not be valid when there is a high amount of subcutaneous body fat, such as obese and overweight people. Furthermore, further studies are necessary to provide answer regarding other body parts.

## 5. Conclusion

To conclude, these data suggest that for the upper arm, both MT and AC are able to detect RT-induced changes in muscle size. Thus, both present useful tools for studies investigating hypertrophic adaptation. However, the magnitudes of change detected by either may be dissimilar, and so care should be taken when comparing estimates between studies and in a combination of effect sizes for the purposes of meta-analysis. Considering the relative ease of use and lesser cost of AC, this might be deemed a desirable approach for researchers with budgetary and equipment limitations, in addition to practitioners and those engaging in RT.
